# Supplementary material for: Transcriptomic and Cytogenetic Analysis of Oxaliplatin-Resistant Colorectal Adenocarcinoma HCT116 Cells to Identify Markers Associated with Platinum Resistance
Source: Int J Mol Sci. 2025 Sep 11;26(18):8869. doi: 10.3390/ijms26188869 (PMC12469909; doi:10.3390/ijms26188869)
Supplement: Supplementary file 1 [file ijms-26-08869-s001.zip › ijms-3819873-supplementary.pdf]

International Journal of
Molecular Sciences

MDPI

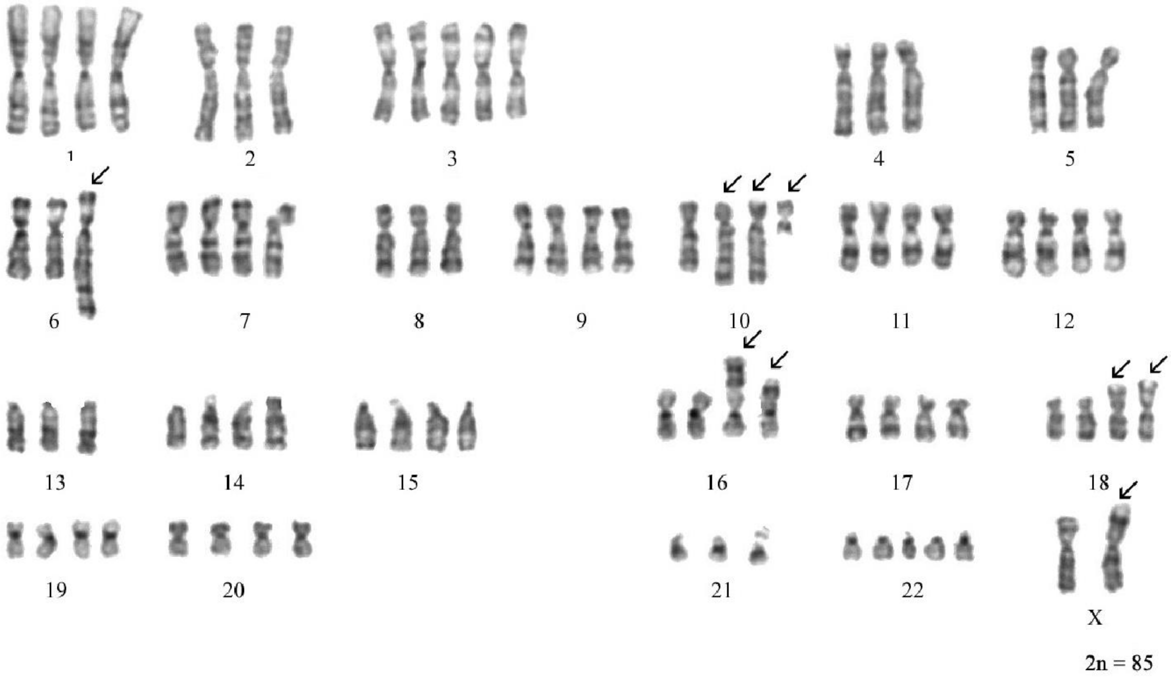

Figure S1. Karyogram of HCT116 oxpl-R cells with 85 chromosomes.

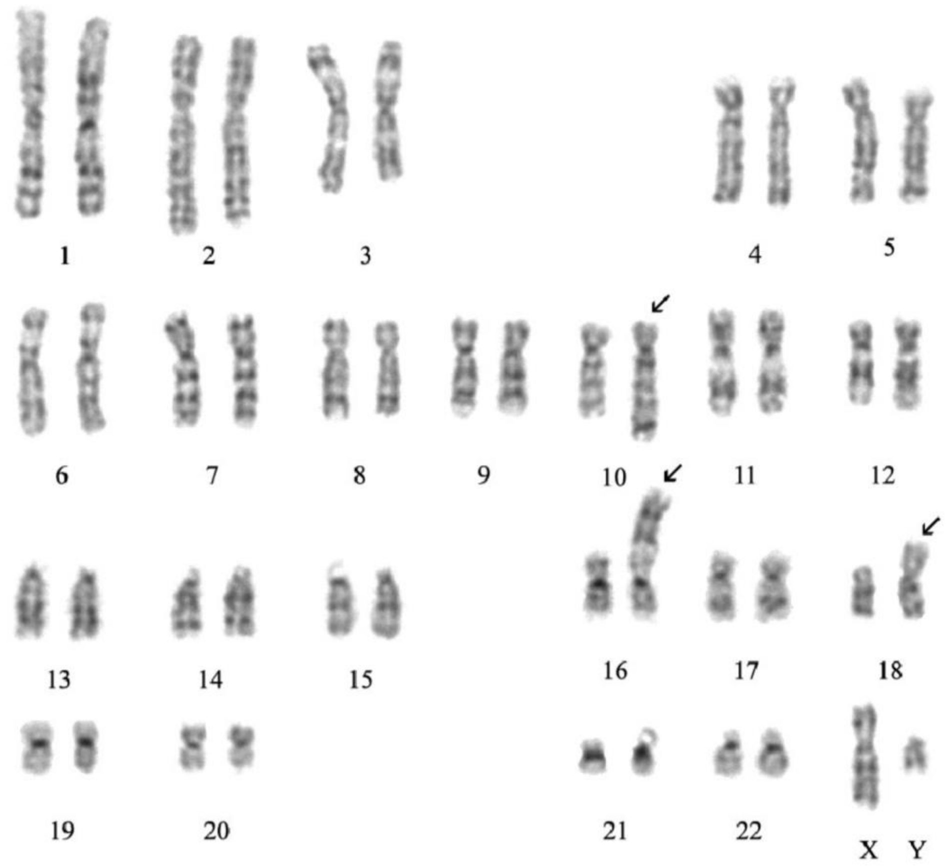

Figure S2: Karyogram of HCT116 cells with karyotype 46,XY,der(10)dup(10)(q22q23),der(8),t(8;16)(q13;p13.3),der(18)t(17;18)(q11.2;p11.2); ***p*****—short arm, q—long arm, der—derivate, dup—duplication, t—translocation.** The arrows indicate the chromosome rearrangements.

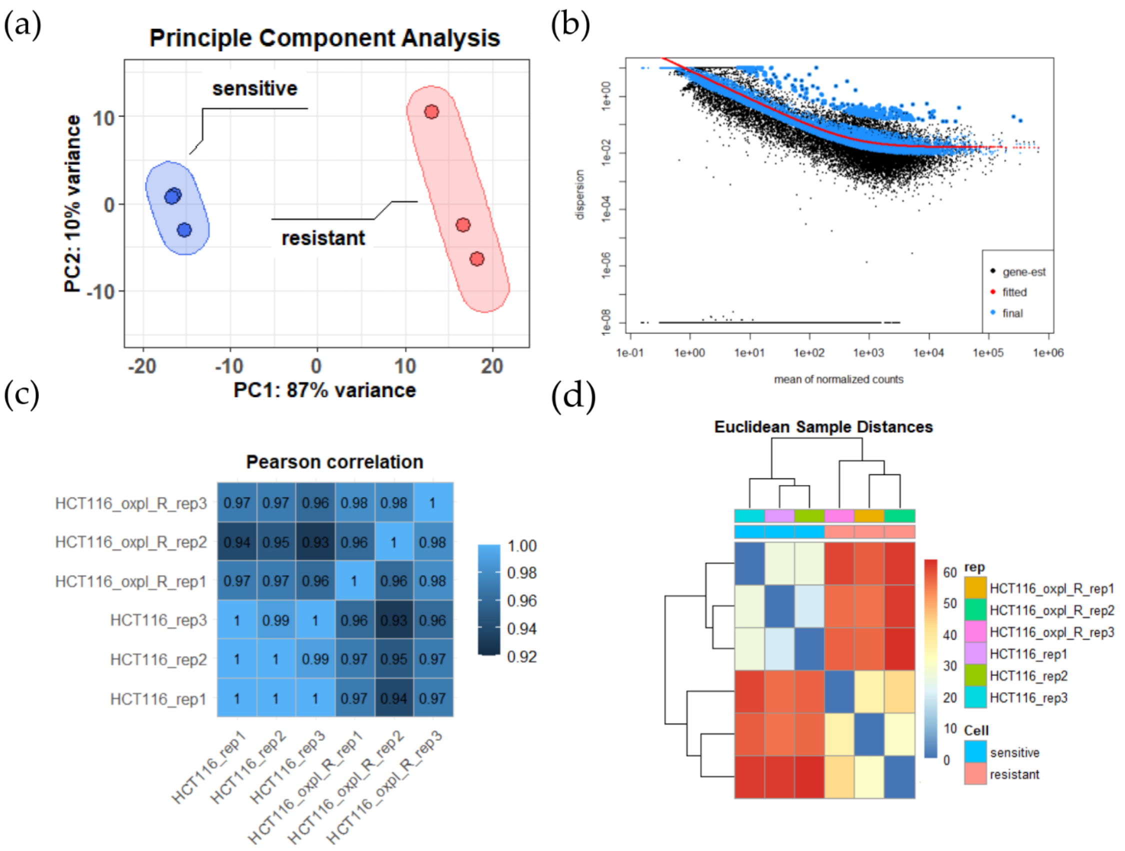

Figure S3. Assessment of inter- and intragroup variability between HCT116 and HCT116 oxpl-R samples. (a) Principal component analysis (PCA) based on the expression profile of HCT116 (blue) and HCT116 oxpl-R (red) cells. The plot displays 6 samples along PC1 and PC2, describing 87% and 10% of the variability, respectively. PCA was applied to normalized read counts. (b) Dispersion estimates plot generated by DESeq2. The plot displays gene-wise dispersion estimates as a function of the mean of normalized counts on a log–log scale. (c,d) Heatmaps displaying Pearson correlation coefficients (c) and Euclidean distances (d) between samples.

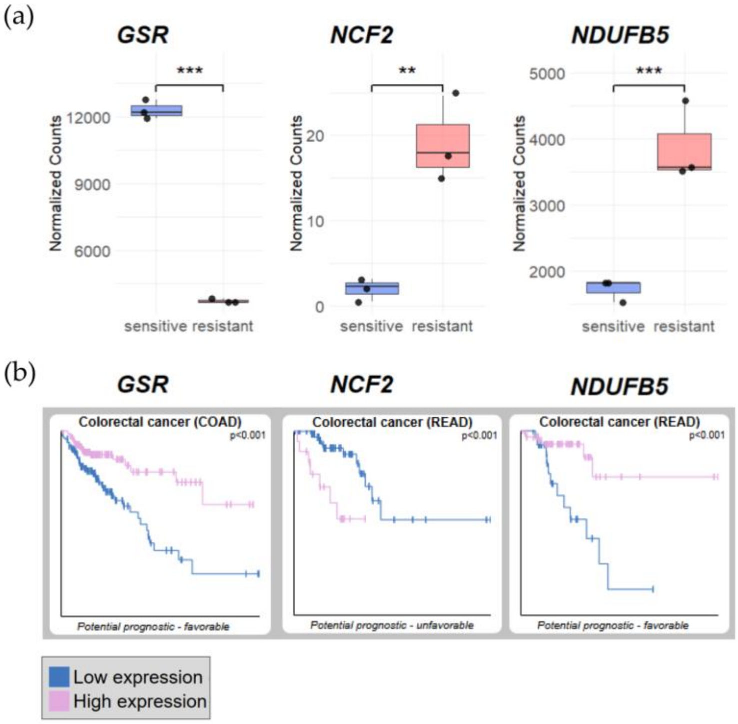

Figure S4: Prognostic value of selected ROS-related genes in colorectal cancer according to the Human Protein Atlas. (a) Boxplots displaying the distribution of normalized counts for selected redox-related genes in HCT116 (sensitive) and HCT116 oxpl-R (resistant) cells, with each point representing a normalized count for an individual sample. The significance shown on the plots is based on the adjusted *p*-values (p.adj) calculated using the Wald test (DESeq2). (b) Kaplan–Meier curves (survival analysis) assessing the correlation between gene expression and survival for colorectal cancer patients (plots downloaded from the Human Protein Atlas, https://www.proteinatlas.org/ (accessed on 2 september 2025**).**
